# Comparison of a Miniaturized Cassette PCR System with a Commercially Available Platform for Detecting *Escherichia coli* in Beef Carcass Swabs

**DOI:** 10.3390/mi12080959

**Published:** 2021-08-13

**Authors:** Dammika P. Manage, Jana Lauzon, Linda M. Pilarski, Patrick M. Pilarski, Lynn M. McMullen

**Affiliations:** 1 Department of Agricultural, Food and Nutritional Science, University of Alberta, 4-10 Ag/For Centre, Edmonton, AB T6G 2P5, Canada; dmanage@ualberta.ca (D.P.M.); jlauzon@ualberta.ca (J.L.); 2 Department of Oncology, University of Alberta and Cross Cancer Institute, 11560 University Ave, Edmonton, AB T6G 1Z2, Canada; lpilarsk@ualberta.ca; 3 5-005 Katz Group Centre for Pharmacy and Health Research, Department of Medicine, Division of Physical Medicine & Rehabilitation, University of Alberta, Edmonton, AB T6G 2E1, Canada; pilarski@ualberta.ca

**Keywords:** lab-on-a-chip, microfluidics, cassette PCR

## Abstract

Detection sensitivity of cassette PCR was compared with a commercial BAX^®^ PCR system for detection of *eae* and *stx* genes in *Escherichia coli* from 806 beef carcass swabs. Cassette PCR detects multiple genetic markers on multiple samples using PCR and melt curve analysis. Conventional PCR served as a gold standard. Overall, for positive and negative concordance, cassette PCR was 98.6% concordant with conventional PCR, and BAX PCR was 65.4% concordant. Of 806 beef carcass swabs, 339 by cassette PCR and 84 by BAX PCR harbored *eae* + *stx+* *E. coli*. For BAX PCR reactions, 84% of *eae*+ swabs, 79% of *stx*+ swabs, and 86% of *eae* + *stx*+ swabs were also detected by cassette PCR. For cassette PCR reactions, 457 swabs were *eae*+ with only 117 scored as *eae*+ using BAX PCR for 26% positive concordance. For *stx* primers, cassette PCR scored 480 samples as *stx*+ but only 215 samples were *stx*+ by BAX PCR, giving 45% positive concordance. Importantly, cassette PCR scored 339 swabs as harboring *eae* + *stx*+ *E. coli*, but BAX PCR detected only 71 positives giving only 21% positive concordance, with many false negatives. Cassette PCR is a highly sensitive method for detection of STEC genes in *E. coli* found in carcass swabs.

## 1. Introduction

Food contaminated with human pathogens is a global health issue. According to the Center for Disease Control and Prevention estimations, 48 million people become ill, 128,000 are hospitalized, and 3000 die each year as a result of foodborne illness. Shiga toxin-producing *E. coli* (STEC) is one of the most prevalent bacterial pathogens in food, especially in meat that can cause severe foodborne disease [1,2]. Detection of pathogens before the food reaches the consumer can prevent outbreaks and save millions of dollars for the food industry by avoiding costly recalls.

Since bacterial pathogens often exist in small numbers in any given quantity of food, an enrichment step is required for preparing samples for essentially all detection systems, in order to increase the number of cells available for detection. Conventional pathogen detection methods such as culture are sensitive and inexpensive but often take 3–5 days. Modern detection methods such as PCR (polymerase chain reaction) and LAMP (Loop mediated isothermal amplification) can provide results with a faster turnaround time and hence often identified as rapid detection methods [3,4]. Among the instruments that are currently available for rapid pathogen detection by PCR is the BAX System Q7 by Dupont Hygiena [5,6,7]. BAX PCR uses dry pelleted PCR reagents with different primer sets for targeting different pathogens. SYBR Green dye that binds to double stranded DNA aids monitoring the progress of PCR process by measuring the fluorescence and also confirms the final product with melt curve analysis (MCA), using a dedicated BAX testing system. The cost of these detection assays and the consumables makes their use in small laboratories prohibitive. Hence cost effective and faster detection methods are still in demand.

With the smaller size, low cost, and rapid testing capabilities, miniaturized lab-on-a-chip devices can change the way food-borne pathogen detection is currently performed [8,9]. We have demonstrated such a device termed “cassette PCR” that is self-contained, simple, disposable, and inexpensive [10,11,12,13,14]. In cassette PCR, PCR is performed in a semi-solid gel, followed by in situ MCA in a capillary reaction unit of ~6 μL volume. This gel contains all reagents including a primer set and Taq polymerase except for the sample to be tested. Each cassette contains array of capillaries with different primer sets, allowing simultaneous detection of multiple targets in parallel. Design and assembly of the cassettes can be altered to the number of the test targets as well as the number of samples. The sample is administered to the ready-made cassette via capillary forces with no pumps or applied pressure allowing the gel to hydrate with the sample. In the assembled cassette, the wax trenches where the capillaries are laid melts during the initial heating of the PCR and hence acts as a vapor barrier as well as segregating capillaries with different primer sets. No other chip sealing techniques are required. The cassette can be stored at room temperature for up to 3 months or longer in the refrigerator [15]. We demonstrated that multiplex PCR can be performed in a given capillary when the gel is desiccated with more than one primer set [14].

Cassette PCR can be used for medical diagnosis, including detection of cancer markers (10), STDs [16], bacteria and viruses [14,17]. Cassette PCR is inexpensive and ready to use. We reported testing of 820 beef carcass swabs for the presence of presumptively pathogenic *E. coli* by cassette PCR, as compared to conventional liquid PCR [13] for carcass swabs that had been collected over a one-year period from provincially inspected processing plants in Alberta by Alberta Agriculture and Forestry (AAF) [18]. The same samples were also tested by AAF using BAX PCR. The main virulence factor of STEC is the production of Shiga toxins, mainly stx1 and stx2. These are considered to be responsible for hemorrhagic colitis (HC) and hemolytic uremic syndrome (HUS) [19,20]. Another virulence factor is intimin, which is an outer membrane adherence protein encoded by the *eae* gene and is responsible for the histopathological lesion called “attaching and effacing” [21]. Therefore, initial screening was performed for the presence of STEC genes (*eae, stx1,* and *stx2*). If the sample scored positive for *eae* plus *stx1* and/or *stx2*, it was considered as presumptively pathogenic. As previously published, cassette PCR testing of these swabs was also performed for high-frequency O-antigens (O157, O26, O45, O103, O111, O121, and O145) [13].

Out of 820 samples, we reported that 41% harbored presumptive pathogens and 30% were O157 positive. Out of these, 19% of samples that were positive for O157 carried potentially pathogenic *E. coli* (*eae* plus *stx1* and/or *stx2*). The samples that were identified pathogenic *E. coli*, 18.9, 38.8, 41.4, 0, 36.1, and 4.1%, were positive for O26, O45, O103, O111, O121, and O145 O-antigens, respectively [13]. Our cassette PCR with all 820 samples were validated with conventional PCR using STEC primers (820 × 4 PCRs). Results showed that only 148 of 3280 cassette PCR tests were discordant with conventional PCR results [13]. Fractional testing of these discordant samples showed that 110 of 148 were the samples with low numbers of *E. coli* in the enrichment broth. Discordance between these samples can be explained as due to Poisson limiting dilution of the template, affecting both cassette PCR and conventional PCR. Out of the remaining 38 discordant tests, 27 initial capillary PCRs and 10 initial conventional tests were nominally discordant between cassette and conventional PCR. These 38 initial results could be due to human/technical error on both sides of the comparison. We concluded that cassette PCR had 98.8% concordance with parallel conventional PCR for detection of STEC genes [13].

In this paper, we compare BAX PCR and cassette PCR testing of 806 beef carcass swab samples for STEC genes *stx1, st2* and *eae*. Pathogenicity of *E. coli* is defined here as detection of both *eae* and *stx* templates in the same sample. Because we have no way to determine whether or not *eae* and *stx* are both present in the same cell, we have considered the designation of pathogenicity to be presumptive. Both BAX and cassette PCR results are evaluated against conventional PCR as a gold standard for sensitivity in detecting STEC. We show that the cassette PCR considerably outperforms BAX PCR in paired studies to detect pathogenic *E. coli* on beef carcass swabs.

## 2. Materials and Methods

### 2.1. Samples

From January–December in 2016, 820 carcass sponge swabs were collected by AAF from seven provincially licensed abattoirs [2 large (>1000 head per year), 3 medium (500–1000 head per year), and 2 small (<500 head per year)]; of these only 806 were available for this part of the study. Protocols for collection and processing the swabs are given in Essendoubi [18]. Briefly, sponge samples were enriched at 42 °C for 15–24 h in modified Tryptic Soy Broth (mTSB, OXOID, Basingstoke, UK). Aliquots of the enriched media were provided for testing by cassette and conventional PCR. Eighty-two batches of enriched samples were received from AAF with the number of samples varied between 2 to 26 per batch. A sterility control (enriched uninoculated media), a positive control (enriched *E. coli* O157:H7 ATCC 35150), and a negative control (enriched media with *E. coli* ATCC 25922) were provided by AAF with each batch. In preparation for the samples for cassette PCR, 8 μL of the enriched sample was added to 72 μL of lysis buffer [13], heated at 55 °C for 15 min followed by 97 °C for 4 min.

### 2.2. Cassette PCR

**Figure 1:**
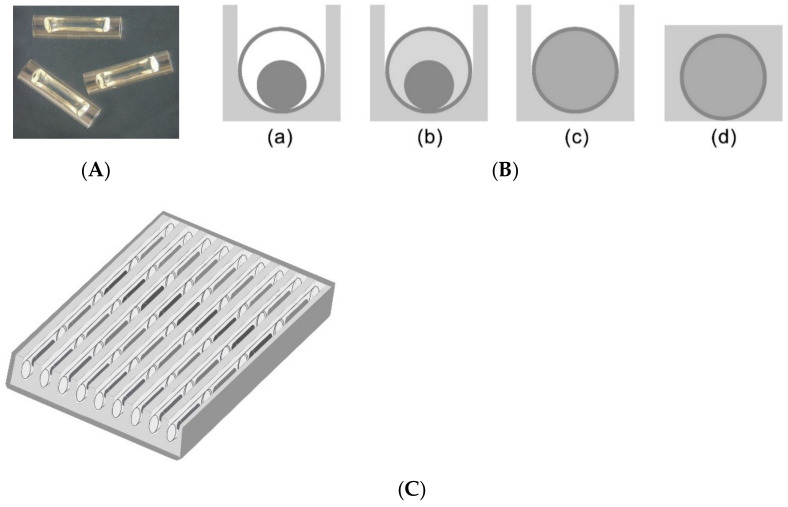
(**A**) Photo of capillaries (6 mm long, 1.1 mm inner diameter, ~6 µL volume) with first polymerized and then dried gel that contains all the ingredients required for PCR. (**B**) Cross-sectional view of a capillary and wax in the cassette (**a**) the desiccated gel inside the capillary before the sample is added; (**b**) after the sample has been added; (**c**) the gel is hydrated; (**d**) capillary after the wax is melted when the cassette is heated during the PCR. (**C**) diagram of the cassette with 9 wax trenches with 4 capillaries with 4 different primer sets in each trench. Each cassette includes internal positive and negative controls.

The cassette used for detecting *E. coli* contained 36 capillaries in 9 trenches with 4 capillaries in each trench containing PCR ingredients and intercalating dye embedded in to a dried gel that includes O157, *eae*, *stx1,* and *stx2* primers in each capillary [13]. Seven out of 9 trenches were used for 7 samples, and the last two were used as negative and positive controls. To each trench with 4 capillary reaction units in the cassette, 25 μL per trench of the processed sample was added to hydrate the gel in each reaction unit. A photo of a capillary is seen in Figure 1A. The space left inside the capillary when the gel is dried allows the sample to flow by capillary forces. Details of the cassette preparation and PCR reaction had been published previously [11]. PCR was performed in a prototype PCR device that runs the PCR and MCA seamlessly via a computer interface [13]. A diagram of the cassette with 36 capillaries is seen in Figure 1C. Briefly, once the PCR begins, wax melts segregating the capillaries to individual reaction units, as seen in Figure 1B. If the primers in the capillary detect the target DNA, PCR amplification starts increasing the number of target segment of double stranded DNA where the intercalating dye, LC Green, binds hence resulting in an increase in the fluorescence of the capillary. The integrated CCD camera takes images of the entire cassette after each cycle is completed.

**Figure 2:**
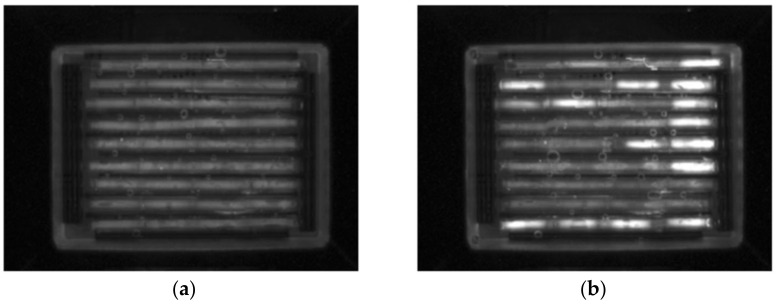
CCD images of a cassette with 36 capillaries in 9 trenches with 4 capillary reaction units per trench that detects 7 swab samples in the first 7 trenches. (**a**) at 1st PCR cycle and (**b**) at 35th cycle. Four capillaries in each trench from left to right have O157, *eae*, *stx1* and *stx2* primers. The last trench (9th) has the positive control and 8th trench has the negative control. These images were first published in Manage et al. [13].

The PCR was performed with a pre-denaturation step of 94 °C for 3 min, then 35 cycles of 94 °C for 15 s, 59 °C for 20 s, and 72 °C for 20 s, followed by a final amplification of 72 °C for 2 min. Examples of CCD images at different PCR cycles are seen in Figure 2. Once the PCR is completed the MCA was run by increasing the temperature from 70 °C to 90 °C and taking the CCD images at 0.2 °C degree intervals.

### 2.3. Conventional PCR

For comparison to cassette PCR, conventional PCR was performed on all 806 samples. Each 25 μL reaction mix consisted of 5 μL of 5xPCR buffer, 0.5 μL of 50 mmol/L MgCl2, 0.5 μL of 10 mmol/L dNTP, 0.3 μL of 2% bovine serum albumin, 0.5 μL of 10 μM primer solution for *stx1* and *stx2*, 1.5 μL of 10 μM primer solution for O157 and *eae*, 0.1 μL of 20 U/mL Taq polymerase, and 6 μL of sample and water. Thermal cycling was performed with a pre-denaturation step of 94 °C for 3 min, then 35 cycles of 94 °C for 20 s, 58 °C for 30 s, and 72 °C for 30 s, followed by a final amplification of 72 °C for 2 min in a thermocycler (Applied Biosystems, Foster City, CA, USA). PCR products were visualized in 2% agarose gels containing SYBR Safe DNA gel stain (Invitrogen, Carlsbad, CA, USA). Primer sequences for *eae*, *stx1,* and *stx2* were published previously [13].

### 2.4. BAX PCR

Methods for sample preparation and PCR were published earlier by Essendoubi et al. [18]. Briefly, 20 μL of the enrichment broth was transferred to BAX lysis buffer and heated to lyse the cells. Thirty µL of this lysate was added to the BAX reaction tubes that contained all the PCR reagents. Real-time PCR was performed in the BAX System Q7 cycler for screening positive samples for the shiga toxin (*stx)*/*eae* pathogenicity genes.

### 2.5. Comparisons among the Three Methods for Detection of Pathogenic E. coli

Volume of broth: BAX PCR tests a volume of 2.72 µL enrichment broth for each sample. Cassette PCR and conventional PCR test only 0.6 µL of enrichment broth for each PCR reaction. Thus, BAX is anticipated to include 4.5 times more target sequences than are available for amplification by cassette or conventional PCR.

Primers: BAX PCR for *stx* genes are preformed using a proprietary mixture of primers for both *stx1* and *stx2*. Cassette PCR and conventional PCR perform separate reactions for *stx1* and *stx2*. For the tables below, these two reaction results are combined to allow comparison with BAX amplification of *stx* genes. It is likely that all primer sets used by BAX are somewhat different from those designed for cassette and conventional PCR, but in positive and negative control broths, all sets were comparable. Because the BAX testing was for *O157:H7* and cassette PCR testing was for O157, we were unable to compare the methodologies for this gene.

For cassette and conventional PCR reactions, all testing runs were always accompanied by positive and negative control capillaries or tubes.

Processing time: Cassette and conventional required 35 PCR cycles, but with conventional PCR there is approximately 1 h required to run PCR products on an agarose gel prior to analysis. RT PCR with BAX completes a total of 55 PCR cycles (personal communication, Hygiena Canada, Mississauga, ON, Canada). The total time from sample input to data acquisition for conventional PCR, cassette PCR, and BAX was 2.5 h, 1.5 h, and 1 h, respectively.

## 3. Results

**Figure 3:**
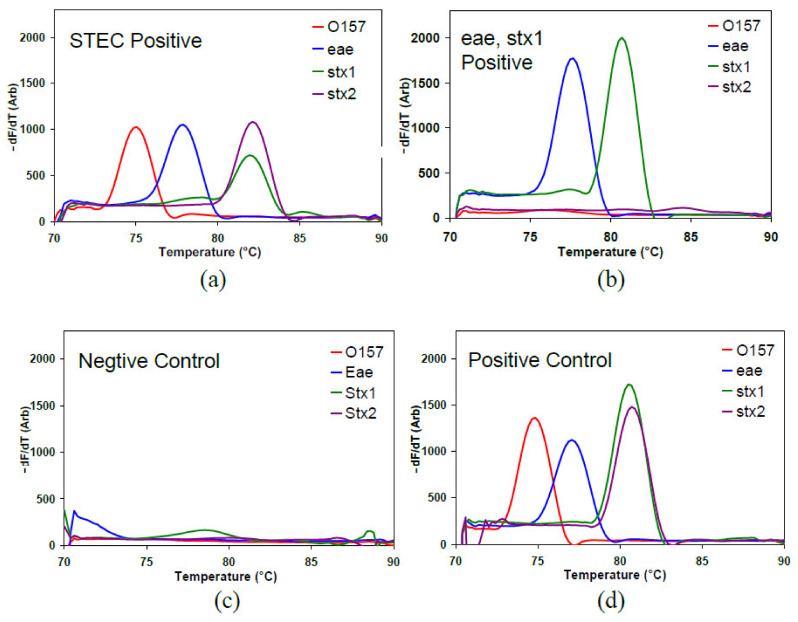
Representation of MCA data of the cassette PCR for two beef carcass swab samples; (**a**) a STEC positive; (**b**) positive for *eae* and *stx1* only; (**c**) the negative control (water); (**d**) the positive control of the cassette. These images were previously published in Manage et al. [13].

In cassette PCR, if the sample contains the target DNA that the primers in the capillary are intended to amplify, the number of double-stranded amplicons increases casing the fluorescence of the capillary to increase with the number of PCR cycles. The capillary is heated during the MCA. Once the melting temperature of the PCR product is reached, the double-stranded amplicon denatures/melts into single-stranded DNA, releasing the intercalated dye molecules and dropping the fluorescence signal abruptly. The melting temperature (T_m_) at which this drop occurs is directly related to the amplicon, which provides a product verification; hence the presence or absence of the target DNA in the given capillary. Plotting the derivative of the fluorescence against the temperature provides an easy visualization of the melting peaks. The MCA data for two samples, a negative and a positive control, are shown in Figure 3 as an example. The presence of a melt peak at the corresponding temperature validates the positivity of the sample to the target pathogenic marker in a given capillary.

BAX PCR data of 806 beef carcass swab samples that were obtained during January-December in 2016 were received from AAF for the *eae* and *stx* primer sets. Cassette PCR/MCA data from the same 806 samples were compared with the BAX PCR data for the same samples, obtained from AAF. Both data sets were compared to the conventional PCR data and concordance and disconcordance between each method are shown Table 1.

Table 1 shows the BAX, cassette, and conventional PCR data for 806 beef carcass swabs. Each box in row 1–4 shows the number of samples scoring positive with the *eae* primer sets (in column 2), *stx* primer sets (column 3), or positive for both *eae* and *stx* primer sets (*eae* + *stx*+, column 4) corresponding to the PCR outcome specified in column 1. Below the number of samples in each box corresponding to a given PCR method are the number of the indicated samples with test results for the other two methods. For example, 140 samples are positive for BAX PCR with *eae* primer set. Out of those 140 samples, 117 samples and 114 samples are also positive by cassette PCR and conventional PCR, respectively. This shows that cassette PCR and conventional PCR have nearly complete positive concordance with each other. While cassette and conventional PCR detect most of the samples positive in BAX testing, there was a major discrepancy in BAX detection of the samples scoring positive by cassette PCR and conventional PCR. Of 339 samples positive for pathogenic *E. coli* by cassette PCR, 331 were also detected using conventional PCR, but only 72 (8.9%) were detected by BAX PCR. Thus, BAX PCR has very low positive concordance with cassette or conventional PCR. Of BAX+ results, only 12 (1.5%, Table 1, Line 7) showed cassette and conventional PCR as non-concordant for positive detection of pathogenic *E. coli* (column 4).

### Overall Positive and Negative Concordance between Cassette PCR, BAX PCR and Conventional PCR

Table 2 shows the outcome if we consider agreement between both positive and negative outcomes of PCR for the three methods. While cassette PCR and conventional PCR are highly concordant in overall results (99%), BAX has a much lower concordance with either cassette or conventional PCR (65–66%), due largely to the number of false negatives.

## 4. Discussion

In this study we compared real-world testing of beef carcass swabs by two PCR methods, the commercially available BAX PCR system and a novel miniaturized system called cassette PCR, with conventional PCR as a presumptive gold standard. Overall, both cassette PCR and conventional PCR were concordant for 795 samples out of 806 samples from beef carcass swabs, among which 331 scored as pathogenic (*eae* + *stx*+) and 464 scored negative. Eleven samples were non-concordant (1.4%) between cassette and conventional PCR. Therefore, the concordance between cassette PCR and conventional PCR is 98.6%. PCR of the 11 samples that did not match could not be repeated, suggesting there may have been a human or technical error during the initial PCR. However, when detecting pathogenic *E. coli*, only 527 samples out of 806 samples agreed between cassette PCR and BAX PCR indicating only 65.4% concordance between the two methods. In this study, compared to conventional PCR as the presumptive gold standard, BAX PCR was considerably less sensitive than cassette PCR.

Conventional PCR is considered one of the gold standards in detecting the presence of a target DNA in a given sample. Overall concordance evaluates both positive and negative agreement between sample sets. Cassette PCR has 96.8% overall concordance with conventional PCR. However, the overall concordance between the conventional PCR and BAX PCR is only 65.7%, reflecting the large number of false negatives. It is not apparent why BAX behaves poorly when detecting the pathogenic *E. coli* samples among the 806 beef carcass swab samples. One possibility is that BAX PCR may be inhibited by components in the enrichment broth, since BAX accepts a larger volume of broth than do cassette and conventional PCR. In BAX PCR, 2.72 µL of undiluted enrichment broth is added in a 30 µL volume of processed sample that is then added to each BAX PCR tube. In cassette PCR, in the 6 µL of processed sample added to each capillary, there is 0.6 µL of undiluted enrichment broth. Therefore, in a given PCR reaction, BAX has ~4.5× more undiluted enrichment broth, and hence 4.5× more pathogen templates as compared to cassette PCR. The presence of enrichment broth as well as other biological matter is known to inhibit the PCR amplification [22,23]. Therefore, the use of smaller volumes of enrichment media in a given PCR may be a benefit. When using crude samples with the presence of enrichment media as well as all the other biological matter present in the swab, the assumed advantage for BAX of adding a larger sample volume with more templates may inadvertently preclude more sensitive detection of pathogenic *E. coli*.

For BAX PCR+ reactions, 84% of *eae* positives, 79% of *stx* positives, and 86% of *eae* + *stx*+ dual positives were also detected by cassette PCR. For cassette PCR+ reactions, with *eae* primers, 457 samples are positive by cassette PCR, of which only 117 samples are also positive for *eae* by BAX PCR with 26% positive concordance. If the *stx* primers are considered, 480 samples are positive by cassette PCR, of which 215 samples are positive for *stx* by BAX PCR with 45% positive concordance. However, of 339 cassette PCR positives for *eae* + *stx*+ presumptive pathogenic samples, BAX detected only 71 positives with 21% positive concordance, indicating that BAX fails to detect most of the samples shown by cassette PCR to harbor presumptively pathogenic *E. coli*.

In summary, the majority of BAX positives were also detected by cassette PCR, but only a minority of cassette PCR reactions that detect a given template(s) were also detected by BAX PCR. Cassette PCR results, but not BAX PCR results, were confirmed by overall and positive concordance with conventional PCR on the same samples. Each sample was tested using all three methods. Therefore, in addition to potential inhibition of BAX PCR reactions by the enrichment broth, the weaker performance in BAX PCR for detecting STEC of pathogenic *E. coli* may also arise from reduced detection with both primer sets, but perhaps to a greater extent from the apparent reduced efficiency of the *eae* primers.

In a study by Bannon 2016, a total of 328 swab samples were collected from hide and de-hided carcasses from two different beef processing facilities in South-Western Ontario over 4 visits within a five-month period [24]. The enriched samples had been screened using RT-PCR GeneDisk system that targeted *stx*, *eae,* and *wzx* genes and found that 92.5% (172 of 186) of the hide samples and 72.5% (29 of 40) de-hided samples scored as presumptive positives. These 328 samples were also tested with BAX PCR for the six most frequent O antigens. For these primer sets, the results from both GeneDisk and BAX PCR agreed very closely. With different target genes from the Bannon study and differing primer sets for *eae* and *stx* genes, the 806 carcass swab samples tested here by cassette PCR and BAX PCR for *eae/stx* positivity scored 42.1% and 10.4%, respectively, failing positive concordance. Thus, it is possible that differing primer sets may contribute to the poor performance and non-concordance of BAX results. This cannot be verified, however, because all BAX primer sets are proprietary. Our observations do raise questions as to the extent to which the incidence of pathogenic *E. coli* may be underestimated by BAX PCR testing for STEC, perhaps dependent upon the nature of the sample material used for BAX PCR.

## 5. Conclusions

We demonstrated a miniaturized cassette PCR device that is self-contained, simple, disposable, and inexpensive, with PCR/MCA performed on a prototype instrument. This device, a gel capillary cassette, contains arrays of capillary reaction units for simultaneously detecting multiple targets. Here, we used cassette PCR for detecting pathogenic *E. coli* in 806 beef carcass swabs that were collected over a one-year period from provincial meat processing plants in Alberta. The overall concordance between cassette PCR and conventional PCR is 98.6%. Of the 84 BAX+ results for *eae* + *stx*+ pathogenicity, 71 (84.5%) showed positive concordance with conventional or cassette PCR. However, for cassette PCR, BAX PCR only poorly agrees, with only 21% positive concordance for detecting STEC of pathogenic *E. coli* (*eae* + *stx*+), due to a high incidence of false negatives. This apparently insensitive detection of pathogenic *E. coli* by BAX PCR may reflect, at least in part, inhibition of BAX PCR by the enrichment broth and/or different primer designs. Overall, our work suggests that cassette PCR, as confirmed by conventional PCR, provides a highly sensitive method for detection of STEC genes in pathogenic *E. coli* found in carcass swabs.

## Figures and Tables

**Table 1:** Comparison between cassette PCR, BAX PCR and conventional PCR (Con. PCR) data with 806 beef carcass swab samples for PCRs performed with *eae* primers (column 2) and *stx* primers (column 3). Column 4 shows the number of indicated swabs for each box that harbored both *eae* and *stx* templates.

PCR Method and Comparison	Number of Positive or Negative Samples		
	*cae*+	*stx* + (*stx1* and/or *stx2*)	*eae* + *stx*+
BAX (+)	140	271	84 (10.4%)
	117 (+) Cassette PCR	215 (+) Cassette PCR	72 (+) Cassette PCR
	114 (+) Con. PCR	214 (+) Con. PCR	71 (+) Con. PCR
Cassette (+)	457	480	339 (42.1%)
	117 (+) BAX PCR	215 (+) BAX PCR	72 (+) BAX PCR
	450 (+) Con. PCR	476 (+) Con. PCR	331 (+) Con. PCR
BAX (−)	666	535	772 (95.8%)
	326 (−) Cassette PCR	270 (−) Cassette PCR	455 (−) Cassette PCR
	329 (−) Con. PCR	267 (−) Con. PCR	459 (−) Con. PCR
Cassette PCR (−)	349	326	475 (58.9%)
	326 (−) BAX PCR	270 (−) BAX PCR	455 (−) BAX PCR
	348 (−) Con. PCR	320 (−) Con. PCR	464 (−) Con. PCR
BAX (−)/Cassette (+)	340	265	267 (33.1%)
BAX (+)/Cassette (+)	117	215	72 (8.9%)
BAX(+)/Cassette (−)	23	56	12 (1.5%)
BAX (−)/Cassette (−)	326	270	455 (56.5%)
BAX(+)/Cassette(+)/Con (+)	78	212	71 (8.8%)
BAX (−)/Cassette (−)/Con (−)	343	266	452 (56.1%)
BAX(−)/Cassette(+)/Con.(+)	372	264	260 (32.3%)
BAX(+)/Cassette(−)/Con.(−)	5	54	12 (1.5%)

**Table 2:** Concordance between cassette PCR, conventional PCR (con.) and BAX PCR.

Method	Cassette−	Cassette+	Concordance
BAX+	72	12	(72 + 455)/806= 65.4%
BAX−	267	455	
	**Con.+**	**Con.−**	**Concordance**
BAX+	71	13	(71 + 459)/806= 65.8%
BAX−	263	459	
	**Cassette+**	**Cassette−**	**Concordance**
Con.+	331	3	(331 + 464)/806= 98.6%
Con.−	8	464	

## Data Availability

Restrictions apply to the availability of these data as some were generated by a 3rd party (Alberta Agriculture and Forestry).
